# Tumour microenvironment: Modulating effects, challenges, and future perspectives of oncolytic virotherapy in Astrocytoma treatment

**DOI:** 10.1016/j.amsu.2022.104508

**Published:** 2022-08-28

**Authors:** Wireko Andrew Awuah, Helen Huang, Jacob Kalmanovich, Aashna Mehta, Mrinmoy Kundu, Abdul Rahman Toufik, Resham Tanna, Mohammad Mehedi Hasan, Athanasios Alexiou, Vladyslav Sikora

**Affiliations:** Sumy State University, Sumy, Ukraine; Royal College of Surgeons in Ireland, University of Medicine and Health Sciences, Dublin, Ireland; Drexel University College of Medicine, USA; University of Debrecen-Faculty of Medicine, Debrecen, Hungary; Institute of Medical Sciences and SUM Hospital, Bhubaneswar, India; Sumy State University, Sumy, Ukraine; Independent Researcher, Chicago, IL, USA; Department of Biochemistry and Molecular Biology, Faculty of Life Science, Mawlana Bhashani Science and Technology University, Tangail, Bangladesh; Department of Science and Engineering, Novel Global Community Educational Foundation, Hebersham, NSW, 2770, Australia; Sumy State University, Sumy, Ukraine

Astrocytomas are the most common type of glioma and arise from astrocytes, which are star-shaped cells found in the cerebrum [1]. Depending on the severity, current therapy include surgical excision, fractionated radiation, chemotherapy, temozolomide, etc [1]. Despite these treatments, the average 5-year survival rate for astrocytomas is only 5% [2]. Oncolytic Virotherapy (OVT) provides a novel treatment that could increase this survival rate and employs viruses to infect and kill tumor cells. Though the use of OVT for malignant melanoma and gliomas in general is well documented, there is a lack of substantial literature to guide the therapeutic effects of OVT in astrocytomas (see Fig. 1).

**Fig. 1:**
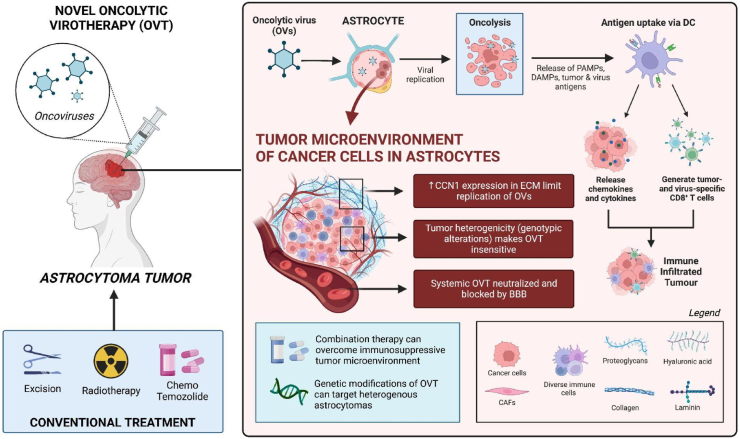
The mechanism of action for Oncolytic Virotherapy (OVT) and the negative influence of the tumour microenvironment, blood-brain barrier, and heterogeneity on the efficacy of OVT. ECM, Extra-cellular matrix; PAMPs, Pathogen-associated molecular patterns; DAMPS, Damage-associated molecular patterns; DC, Dendritic cells; GBM, Glioblastoma multiforme; CAFs, Cancer-associated fibroblasts; CCN1, Cysteine-rich 61; BBB, Blood-brain barrier.

Numerous studies and trials have been conducted to investigate the potential benefits of oncolytic viruses in brain tumors, showing very promising results. Passaro et al. used a modified HSV-1 virus that also encoded an anti-PD-1 antibody in a trial on glioblastoma multiforme (GBM) [3]. GBM, also known as grade IV astrocytoma, has high levels of PD-1, an immune checkpoint protein that, when suppressed, may promote a more effective immunological response from the host. Similarly, clinical trials have deployed a modified virus known as M032 in patients with anaplastic astrocytoma and discovered that OVT caused tumor cells to release interleukin 12 (IL-12) prior to death [4]. These trials demonstrate potential therapeutic benefits that OVT can achieve with additional research. However, little is known about the efficacy of oncolytic viruses in pediatric brain tumors, with astrocytomas being the most common. *In vitro* models of xenograft mouse models injected with fresh surgical samples of anaplastic astrocytoma were administered the seneca valley virus (SVV-001) and shown to infect, replicate, and kill self-renewing glioma cells. However, the therapeutic benefits of OVT in pediatric subjects are mixed in opinion [5]. While Csatary et al. saw significant regression of recurrent glioblastoma multiforme in children after the administration of the newcastle disease virus (MTH-68/H strain) [6], Wagner et al. reported a child with anaplastic astrocytoma who unfortunately passed away after OVT combination with valproic acid failed to regress the primary tumor [7].

There are multiple mechanisms of actions for oncolytic virotherapy documented in the literature, and is attributed to oncolytic viruses preferentially binding and lysing tumor cells [8,9]. However, oncolytic viruses can interact various immune pathways (i.e. IFN pathway) and cause the release of tumor associated antigens, cytokines and chemokines which can promote antigen-specific host immune response to eradicate tumor cells [2]. Of importance is the tumor microenvironment, which is a highly dependent factor that dictates the ability of viruses to infect tumor cells. The tumor microenvironment includes host stromal cells such as fibroblasts, the extracellular matrix (ECM), and different immune cells [10]. In astrocytomas, the environment is often ‘cold’ as it highly expresses immunosuppressive cytokines (i.e. TGFb, IL-10) to inhibit immune eradication and contribute towards its resistance against conventional chemotherapy, radiotherapy, and immunotherapy [11]. OVT was an attractive therapeutic option for the brain due to its ability to recruit tumor infiltrating lymphocytes and cytotoxic T-cells in the tumor microenvironment, thereby eradicating the natural immunosuppressiveness of astrocytoma. This can be attributed to the OVT initiating the release of tumor associated antigens and viral pathogen-associated molecular patterns upon tumor cell lysis [12].

Despite the promising benefits of OVT, we discuss two major barriers that may attribute to the low rate of success in specifically regressing astrocytomas. Firstly, the high heterogeneity of astrocyte tumor microenvironments makes OVT insensitive to certain parts of the tumor [12]. Pediatric brain tumors are highly heterogeneous and often account for its high mortality rate in children. Astrocytomas have substantial differences in its genotypic alterations and phenotypic expression, making targeted viral replication in the tumor microenvironment difficult [13]. Moreover, its diverse expression of IFN in the microenvironment can alter responses of the anti-viral pathway, insensitive to OVs [14]. Though systemic therapy has been suggested to stimulate anti-tumor responses and overcome the heterogeneity of tumor associated antigens, this mode of delivery is severely hampered by the neutralization and blockage of viruses [15,16]. Second to heterogeneity is the resistance of treatment due to the extracellular matrix (ECM). The connective tissue and ECM is well known to inhibit the spread of OVs and contributes to the survivability and invasiveness of brain cell tumors, but it is difficult to ascertain specific components that cause OVT insensitivity within astrocytomas. Cysteine rich 61 (CCN1) is a protein that is tightly associated with the extracellular matrix in cancer cells and was found: 1) promote cell growth in glioma cells, and 2) orchestrate cellular antiviral responses that limit the replication of OVs [17]. In astrocytes, it was found that one of its gap junction proteins (Cx43) upregulated the expression of CCN1 and may attribute to the aforementioned characteristics [18]. Given the association of CCN1 expression in astrocytes and its role in the ECM, it is reasonable to infer that the lack of OVT sensitivity in astrocytomas is due to antiviral responses exhibited by the proteins in the ECM and may need to be further validated in future studies.

The paucity of literature surrounding the challenges of OVT in astrocytoma hinders effective recommendations that may overcome OVT insensitivity. It is clear that its performance as a monotherapy, though proven safe, is not efficacious. Combination therapy with a variety of immunotherapies such as immune checkpoint inhibitors, immunotherapeutic modulators, anti-tumor vaccines, and adoptive cell therapy are all promising avenues in overcoming the immunosuppressive microenvironment of astrocytomas and supported in current clinical trials [19]. However, the genetic modifications of OVs are emerging as a viable option to improve the specificity of OVT and overcome the aforementioned challenges. A great summary of the applicability of genetically modified OVs can be found in Cristi et al., where genetic modifications can promote viral replication in tumors, overcome the ECM barrier, and stimulate various signaling pathways that would effectively target the heterogenic tumor microenvironment [20]. Further studies exploring OVT as a primary treatment option and its synergism with other agents can help demonstrate its efficacy alone and as a supplement. Moreover, further investigations on the efficacy of OVT in pediatric brain tumors and the role of predictive biomarkers in determining OVT response should be prioritized especially due to the heterogeneity of astrocytomas [21]. Therefore, further research aimed at identifying potential targets, such as ECM proteins and IFN pathways, to reduce immune response generation against OVT and potentiate its effect can help optimize OVT and achieve maximal therapeutic outcomes in Astrocytomas.

## Please state any conflicts of interest

N/A.

## Ethical approval

N/A.

## Please state any sources of funding for your research

N/A.

## Consent

N/A.

## Author contribution

WAA, AM, HH, AA Conceptualized the topic, coordinated reading, writing and editing:

WAA, AM, JK, HH, MK, AT,RT, and MH contributed to reading, writing, editing the original draft and critical revision: JK, MK, and MMH contributed to various aspects of reading, data collection, writing the original draft and implementing changes for critical revision under the supervision of WAA, AM, HH, AA, and VS.

## Registration of research studies

1. Name of the registry: N/A
2. Unique Identifying number or registration ID: N/A
3. Hyperlink to your specific registration (must be publicly accessible and will be checked): N/A

## Guarantor

Mohammad Mehedi Hasan.

Department of Biochemistry and Molecular Biology, Faculty of Life Science, Mawlana Bhashani Science and Technology University, Tangail, Bangladesh; mehedi.bmb.mbstu@gmail.com (MMH).

## Declaration of competing interest

N/A.
